# Transmission of Multidrug-Resistant Salmonella enterica Subspecies enterica 4,[5],12:i:- Sequence Type 34 between Europe and the United States

**DOI:** 10.3201/eid2612.200336

**Published:** 2020-12

**Authors:** Ehud Elnekave, Samuel L. Hong, Seunghyun Lim, Dave Boxrud, Albert Rovira, Alison E. Mather, Andres Perez, Julio Alvarez

**Affiliations:** Hebrew University of Jerusalem, Jerusalem, Israel (E. Elnekave);; University of Minnesota, St. Paul, Minnesota, USA (E. Elnekave, S. Lim, A. Rovira, A. Perez, J. Alvarez);; University of Leuven, Leuven, Belgium (S.L. Hong);; Minnesota Department of Health, St. Paul (D. Boxrud);; Quadram Institute Bioscience, Norwich, UK (A.E. Mather);; University of East Anglia, Norwich (A.E. Mather);; Universidad Complutense, Madrid, Spain (J. Alvarez)

**Keywords:** Evolution, *Salmonella* monophasic, Salmonella enterica subspecies enterica, serotype 4,[5],12:i:-, multidrug-resistance, antimicrobial resistance, foodborne, zoonotic, zoonoses, enteric infections, bacteria, Europe, United States

## Abstract

Multidrug-resistant *Salmonella* *enterica* subspecies *enterica* 4,[5],12:i:- sequence type 34 represents a worldwide public health risk. To determine its origin in the United States, we reconstructed a time-scaled phylogeny with a discrete trait geospatial model. The clone in the United States was introduced from Europe on multiple occasions in the early 2000s.

Since the late 1990s, reports of an emerging multidrug-resistant *Salmonella enterica* subspecies *enterica* serotype 4,[5],12:i:- strain have been published in Europe (*1*). This strain is a monophasic variant of *Salmonella* Typhimurium, predominantly resistant to ampicillin, streptomycin, sulfonamides, and tetracycline (ASSuT). Its rapid increase in North America after 1998 has also been described (*2*). However, precise knowledge of the time of introduction and the initial influx of clinical cases caused by this serotype in the United States is not available because of inconsistent reporting before 2004 (*3*).

Previously, on the basis of high genetic similarity between *Salmonella* 4,[5],12:i:- sequence type (ST) 34 isolates from the United States and Europe and *Salmonella* Typhimurium strains from Europe, we suggested a European origin for the *Salmonella* 4,[5],12:i:- ST34 clade (*4*). With this study, we aimed to reconstruct a time-scaled phylogeny of the emerging ST34 clade by using a Bayesian modeling approach to determine its origin in United States.

## The Study

**Figure 1:**
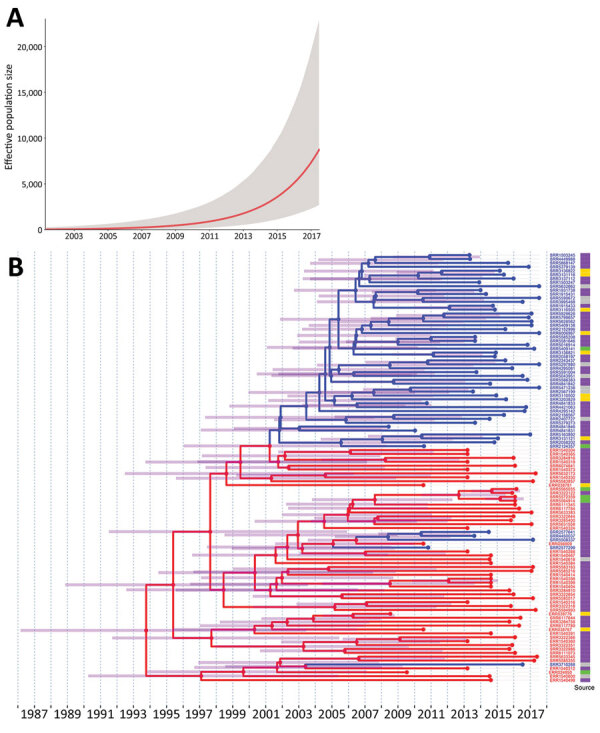
Demographic reconstruction and phylogenetic analysis of *Salmonella* *enterica* subspecies *enterica* 4,[5],12:i:- sequence type 34 isolates. A) Demographic reconstruction (subset 2) shows the population exponential growth over time. The red line indicates the median effective population size with 95% highest posterior density credible interval (gray). B) Time-scaled phylogenetic analysis of isolates in subset 2 (n = 110 sequences after duplicates removal). Isolates were collected from multiple sources in the United States (blue) and Europe (red) during 2008–2017.An asymmetric discrete trait analysis model was used to reconstruct the locations on the nodes. The nodes, branches, and tree tips were annotated according to the collection location. The 95% highest posterior density credible intervals of node heights are indicted with transparent purple bars. The posterior probability for all inferred ancestral locations was >70%. The isolate source (food product, gray; human, purple; livestock, yellow; and other, green) is depicted in the heatmap appended to the tree tips.

**Figure 2:**
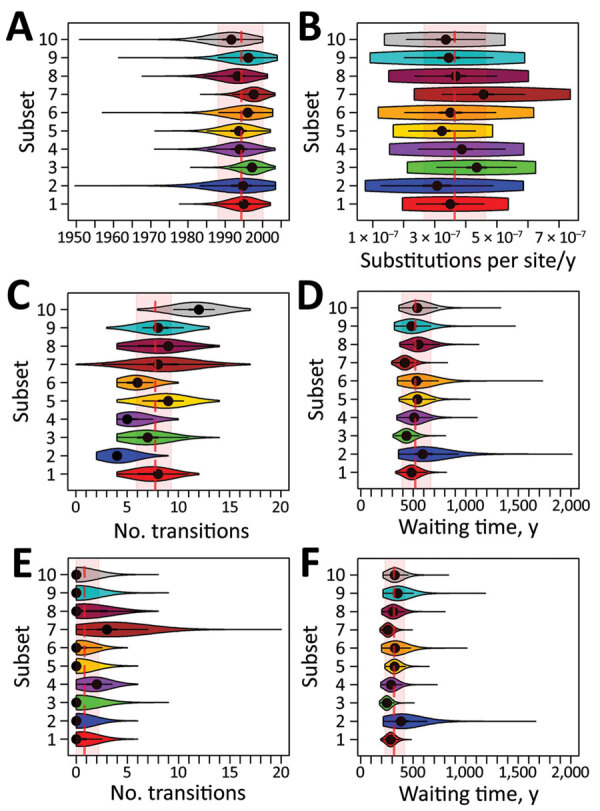
Summary of posterior estimates of all 10 subsets of sequences of *Salmonella* *enterica* subspecies *enterica* 4,[5],12:i:- sequence type (ST) 34 collected from multiple sources in the United States and Europe during 2008–2017. A) Inferred time (year) of the most recent common ancestor of the emerging *Salmonella* 4,[5],12:i:- ST34 clade. B) Estimated mutation rate (uncorrelated log-normally distributed mean parameter). C–F) Number of unobserved transitions from Europe to the United States (C) and United States to Europe (E) along each branch (Markov jumps) and total phylogenetic tree length spent (Markov rewards) in Europe (D) and the United States (F). Violin plots illustrate the posterior distribution and probability density of each subset. Dashed red vertical lines indicate average posterior value; red shaded areas indicate average 95% highest posterior density credible interval of all subsets.

We used publicly available whole-genome sequences of 1,431 *Salmonella* 4,[5],12:i:- ST34 isolates from the United States and Europe from 2008–2017, including sequences from 690 isolates from Europe (mainly from the United Kingdom and Denmark) and 741 isolates from multiple US states (Appendix 1). We used BEAST version 1.8.4 (*5*) to estimate divergence times, mutation rates, and location trait transitions. We applied the modeling approach to 10 subsets of 112 sequences selected from the study population. These sequences represented 33% (474/1,431) of the study population and included 242 sequences from Europe (76% from humans, 8% from food products, 8% from livestock, and 8% from other sources) and 232 from the United States (62% from humans, 13% from food products, 21% from livestock, and 3% from other sources). Time-scaled phylogenies of each subset were reconstructed by using a general time-reversible nucleotide substitution model, an uncorrelated lognormal relaxed molecular clock, and an exponential growth coalescent model with asymmetric trait transitions (Figure 1; Appendix 2 Figures 1–10). All time-scaled phylogenies presented similar topology to a maximum-likelihood phylogeny constructed by using all 1,431 study isolates (based on visual inspection; Appendix 2 Figure 11). Overall, averaged estimates from all subsets were in agreement as follows (Figure 2): the evolutionary rate was 3.64 × 10^−7^ substitutions/site/year (95% highest posterior density [HPD] 2.65–4.64 × 10^−7^), which corresponds to an accumulation of »1–2 single-nucleotide polymorphisms/genome/year; the time to most recent common ancestor was 1994 (95% HPD 1988–2000); the number of collection location state transitions (Markov jumps) from Europe to the United States was 7.7 (95% HPD 5.9–9.3) and from the United States to Europe was 0.8 (95% HPD 0–2.2); and the waiting times (in years; Markov rewards) were 519.9 (95% HPD 393.1–667.8) in Europe and 318.6 (95% HPD 234.0–417.6) in the United States. The exponential growth rate of the population was estimated at 0.316/year (averaged across all subset means; Figure 1). In addition, the estimated (averaged) main introduction into the United States was 2004 (95% HPD 2000–2006; Appendix 2 Table 1). The occurrence of several additional smaller introductions was suggested by 48 sequences (6 from Europe and 42 from the United States). The 6 sequences from Europe were recovered from human sources; among sequences from the United States, 28 were from humans, 5 from food products, 6 from livestock, and 3 from other sources. Information on recent international travel was obtained for 22/28 of US isolates from humans, 2 of whom had traveled (1 to the Philippines and the other to France [S. Meyer et al., Minnesota Department of Health, pers. comm., 2019 Sep 23]).

Among the 1,431 *Salmonella* 4,[5],12:i:- ST34 sequences, 978 (68.34%) had genetic determinants contributing to the ASSuT profile, 108 (7.55%) conferred resistance to quinolones, and 82 (5.73%) conferred resistance to extended-spectrum cephalosporins. The probability of harboring most predominant acquired antimicrobial resistance genes (AARGs) conferring the resistance phenotypes described above was significantly higher for sequences of US isolates (odds ratio 2.37–26.05; Table). Yet associations between the collection location and the presence of *bla*_CTX-M_ or *qnrS1* genes were not significant (Table). In addition, AARGs conferring resistance to colistin (*mcr*-1/*mcr*-3/*mcr*-5; Appendix 1) were detected in isolates from Europe only (n = 5).

**Table:** Association between collection location and presence of resistance genetic determinants in sequences of *Salmonella enterica* subspecies *enterica* serotype 4,[5],12:i:- sequence type 34 isolates collected in Europe and the United States, 2008–2017*

Conferring resistance to	Presence of genetic resistance determinants	No. positives/total (%)		Odds ratio (95% CI), United States vs. Europe	p value†
		Europe	United States		
ASSuT	ASSuT‡	406/690 (58.84)	572/741 (77.19)	2.37 (1.87–3.00)	**<0.001**
Extended-spectrum cephalosporins	*bla*_CTX-M_ genes§	4/690 (0.58)	14/741 (1.89)	3.30 (1.03–13.84)	0.032
	*bla*_CMY-2_	2/690 (0.29)	37/741 (4.99)	18.06 (4.63–155.09)	**<0.001**
	*bla*_SHV-12_	0/690¶	27/741 (3.64)	53.15 (3.24–873.11)	**<0.001**
Quinolones	*qnrB19*	9/690 (1.3)	51/741 (6.88)	5.59 (2.70–13.01)	**<0.001**
	*qnrB2*	0/690¶	20/741 (2.7)	39.24 (2.36–650.05)	**<0.001**
	*qnrS1*	6/690 (0.87)	13/741 (1.75)	2.03 (0.72–6.57)	0.22
	*aac(6¢)-Ib-cr*	1/690 (0.14)	19/741 (2.56)	18.11 (2.86–751.91)	**<0.001**

*ASSuT indicates ampicillin, streptomycin, sulfonamides, and tetracycline. †A statistically significant *p value* (boldface) is <0.05/8 = 0.00625 (adjusted for multiple comparisons using Bonferroni’s correction). ‡Simultaneous presence of *bla*_TEM-1_, *strA*, *strB*, *sul2*, and *tet(B)* genes (Appendix 2). §Including *bla*_CTX-M-1_ (n = 1), *bla*_CTX-M-14_ (n = 2), *bla*_CTX-M-55_ (n = 14), and *bla*_CTX-M-65_ (n = 1). ¶Haldane-Anscombe correction (adding 0.5 to all 4 cells) was used to account for cells with a value of 0.

## Conclusions

*Salmonella* 4,[5],12:i:- ST34 was introduced into the United States from Europe on multiple occasions since the beginning of the 21st century. The main introduction occurred in 2004; additional independent introductions resulted in small clades for which the predominant sources were human travelers and imported food products. Human travelers (*6*) and imported food products (*7*) have been described as potential vehicles for introduction of salmonellae.

The date of introduction of the main clade into the United States is later than the first peer-reviewed report of a *Salmonella* 4,[5],12:i:- infection in the country in 1998 (*2*). However, given the antimicrobial susceptibility profile of isolates from that report (mostly not ASSuT) (*2*), they most likely belonged to the nonemerging ST19 clade, which was described elsewhere (*4*). In addition, the incidence of *Salmonella* 4,[5],12:i:- in humans increased only modestly (9.5%) during 2006–2011 but increased dramatically (78.3%) during 2011–2016 (*8*). A similar increase in detection after 2011 was described for clinical cases in swine from the midwestern United States (*9*). The difference between the date of main introduction into the United States found in this study and the later sharp increase in its prevalence in animals and humans may in part result from changes in reporting practices and increasing awareness (*8*). However, the increase since 2011 can be the result of rapid propagation of the ST34 population, possibly associated with swine (*4*). Moreover, White et al. (*10*) recently reported that according to the National Antibiotic Resistance Monitoring System, the percentage of ASSuT-resistant *Salmonella* 4,[5],12:i:- from humans increased from 17% in 2009 to 59.1% in 2015 (out of all *Salmonella* 4,[5],12:i:- clinical isolates from humans). This increase probably resulted to a large extent from ST34 strains, in which this phenotype is predominant. The estimated exponential yearly growth rate determined in our model (0.316/year), which corresponds to a population doubling time of 2.2 years, is in agreement with this dramatic increase of the ST34 population.

The presence of AARGs conferring resistance to quinolones and extended-spectrum cephalosporins has mainly been observed since 2014 and may be biased by the lack of sequences before 2013 (Appendix 2 Figure 12). Yet AARGs conferring resistance to quinolones were not found in *Salmonella* 4,[5],12:i:- ST34 strains from Europe collected before 2010 (*1*), and therefore our findings may reflect an increasing prevalence of these resistance determinants. Given time and overall unidirectionality of *Salmonella* 4,[5],12:i:- ST34 transmission from Europe to the United States, it is likely that the acquisition of AARGs to quinolones occurred independently in the United States and in Europe. Yet introduction of resistant strains from the United States to Europe is also possible. Contributors to the acquisition of resistance in the United States might be the approval for enrofloxacin use in swine in the United States since 2008 (*11*) and the potential dissemination of plasmids harboring AARGs to quinolones between *Salmonella* serotypes (*12*). Independent acquisition of resistance to quinolones by *Salmonella* in Asia has also been suggested (*13*). The presence of *mcr* resistance genes conferring resistance to colistin in sequences from Europe (n = 5) is alarming, given their recent worldwide spread (*14*). However, further investigation of the travel history associated with these cases may be required because the acquisition of *mcr* genes may be travel associated (*15*). The spread of *Salmonella* 4,[5],12:i:- ST34 from Europe to the United States and the presence of plasmid-mediated resistance genes to key antimicrobial classes such as quinolones, extended-spectrum cephalosporins, and colistin in this clade further highlights its potential risk to public health and emphasizes the need for robust surveillance and mitigation programs for such transboundary pathogens.

Appendix 1 Dataset of 1,431 publicly available *Salmonella enterica* subsp. *enterica* 4,[5],12:i:- sequence type 34 isolates from Europe and United States, 2008–2017.

Appendix 2 Supplemental methods and results for study of transmission of multidrug-resistant *Salmonella enterica* subsp. *enterica* 4,[5],12:i:- sequence type 34 between Europe and United States, 2008–2017.
