# Supplementary material for: Transmission of Multidrug-Resistant Salmonella enterica Subspecies enterica 4,[5],12:i:- Sequence Type 34 between Europe and the United States
Source: Emerg Infect Dis. 2020 Dec;26(12):3034–8. doi: 10.3201/eid2612.200336 (PMC7706949; doi:10.3201/eid2612.200336)
Supplement: Appendix 2 — Supplemental methods and results for study of transmission of multidrug-resistant Salmonella enterica subsp. enterica 4,[5],12:i:- sequence type 34 between Europe and United States, 2008–2017. [file 20-0336-Techapp-s2.pdf]

Article DOI: https://doi.org/10.3201/eid2612.200336

Transmission of Multidrug-Resistant *Salmonella enterica* Subspecies *enterica* 4,[5],12:i:- Sequence Type 34 between Europe and the United States

Appendix 2

Study population

The metadata of 1431 *Salmonella enterica* serotype 4,[5],12:i:- of sequence type (ST)34 isolates collected in USA (from multiple states) and Europe (mainly from United Kingdom and Denmark; TableS1) from 2008 through 2017 (excluding years 2011 and 2012) was obtained through various public sources (see below). Overall, the analysis included sequences of 690 European isolates and 741 isolates from USA (of which 73%, 10%, 4% and 13%, and 61%, 15%, 20% and 4% were recovered from humans, food products, livestock and other sources in Europe and USA, respectively). Availability of sequences from both USA and Europe on a certain collection year was set as a selection criterion to prevent potential bias of representing historic samples from a single location. In addition, when available, the sequences full collection date was used. Mid-year or mid-month were used as proxy when only the year or the year and month of collection were available, respectively. The study sequences were mainly identified using the National Center for Biotechnology Information (NCBI) 'pathogen detection' platform (https://www.ncbi.nlm.nih.gov/pathogens/; accessed: October 2017) (n=1162/1431). Additional sequences were found using the European Bioinformatics Institute (EMBL-EBI; https://www.ebi.ac.uk/) website (n=196/1431) and a recent study on *S*. 4,[5],12:i:- in USA (n=73/1431) (*1*).

Similar number of samples from each location-year combination were analyzed to avoid possible temporal and/or spatial selection biases that could affect the outcome of the analysis. Therefore, 50 sequences from years in which sequences were available from only one location were excluded from the analysis. This has further decreased the number of *S*. 4,[5],12:i:- ST34

sequences that were collected before 2013 and were available in the NCBI repository (Appendix 2, Figure 12), which may have limited the temporal signal in the dataset and biased the sample selection for the different subsets. Moreover, with the exception of *S*. 4,[5],12:i:- ST34 sequences from Vietnam, for which a European origin was previously suggested (*2*), there is limited availability of *S*. 4,[5],12:i:- ST34 sequences from world regions other than USA and Europe (mainly from United Kingdom and Denmark), and from non-human samples. Therefore, we could not assess the importance of other locations (different from USA and Europe) or to demonstrate the transmission of the pathogen between sources, respectively. However, despite these limitations, we were able to include 33% of the available *S*. 4,[5],12:i:- ST34 study population initially considered and to consistently demonstrate in multiple random subsets the possible directionality of transmission from Europe into USA (see text). The agreement with a preceding study in which a similar conclusion was obtained based on genetic similarities between *S*. Typhimurium and its monophasic variant from USA and Europe (*1*) further supports our findings.

Data analysis

Time-scaled phylogeny and discrete trait analysis

Bayesian Evolutionary Analysis Sampling Trees (BEAST v1.8.4 (*3*)) was used for estimation of divergence times, mutation rates and location trait transitions.

Subsets selection

Ten subsets of 112 sequences were selected for a time scaled analysis. To minimize the impact of potential spatial and temporal biases on the analysis outcome, the data was first stratified by collection location (Europe and USA) and by collection year. Then, an equal number of sequences from each location-year combination were randomly selected from the available sequences (up to ten sequences from each location-year combination) for each subset. Overall 474/1431 sequences were included in the selected subsets, of which 129 (27.2%), 233 (49.2%), 43 (9.1%), 35 (7.4%), 13 (2.7%), 7 (1.5%), 8 (1.7%), 1 (0.2%) and 5 (1.1%) were represented in a single, two, three, four, five, six, seven, eight and ten of the subsets, respectively.

Data filtering and quality control

Paired-end Illumina reads were downloaded from the NCBI repository and read quality was assessed using FASTQC (v0.11.6 (*4*)). De novo genome assemblies were constructed using the SPAdes assembler (v3.12.0) with the '--careful' option (*5*)). When necessary, the 'repair.sh' command in BBmap (*6*) was used to fix disordered raw reads before reassembly. The quality of contigs was assessed using QUAST (v4.6.3 (*7*)). Only sequences for which the N50 of assembled contigs was at least 30,000 nucleotides were further analyzed. SRR3322114 had a low N50 (10,567) yet was mistakenly included in the analysis (subsets 5 and 7). However, the inclusion of this single sequence is unlikely to affect the outcome, as also demonstrated by the similar outcomes of the multiple subsets used. In addition, the *Salmonella* In Silico Typing Resource (SISTR) v1.0.2 (*8*) was used only on 67% of the assemblies. However, we found that 96% of the in silico predicted serotype agreed with the reported serotype (the remaining 4% were removed from the analysis). Therefore, the represented population consisted of mainly 4,[5],12:i:- and not the diphasic ST34. In addition, the quality of all contigs from the *de novo* genome assemblies of the selected subsets (n=474 sequences) was further assessed: Bowtie2 (v2.3.4.1 (*9*)) was used to align the raw reads to the contigs and BBmap (v38.06 (*6*)) was used to calculate the average coverage depth of the contigs. Only sequences in which the average coverage depth of the contigs was at least 20 were included in further analysis.

Temporal signal

Reconstruction of maximum likelihood (ML) phylogeny trees for each subset was conducted for a priori estimation of the 'temporal signal' of the data. Raw reads were mapped to the reference *S.* 4,[5],12:i:- genome strain S04698-09 (RefSeq NZ_LN999997) using bowtie2 (v2.3.4.1 (*9*)) with default parameters. SAM files were compressed into BAM and sorted using 'view' and 'sort' commands from SAMtools (v1.3 (*10*)). Candidate single nucleotide polymorphisms (SNPs) were identified by using SAMtools (v1.3 (*10*)) and VarScan2 (v2.3.9 (*11*)). For each BAM alignment, a pileup file was created using 'mpileup' command from SAMtools (*10*), and only high quality SNPs were selected using 'mpileup2snp' command from VarScan2 (*11*) with the following criteria: a coverage threshold of 8, a minimum average base quality (Phred quality score) of 30, and a 90% agreement threshold. SNPs found using this method were output in variant call format and were parsed and examined. Phage regions were detected in the reference genome using PHASTER (*12*) and SNPs within such regions were

masked using a custom python script (below – script 1). Recombination regions were identified using Gubbins (v2.3.2 (*13*)). Duplicate sequences (i.e. different isolates but genetically identical) were removed using a custom python script (below –script 2). Phylogenetic ML tree reconstruction was conducted for each subset separately using RAxML (v8.2.10 (*14*)) with the following definitions: a general time reversible substitution evolutionary model with gamma correction (GTR+Γ), 250 rapid bootstrap replicates and four randomly generated rapid bootstrap seeds. The best tree was selected based on the maximum likelihood and all bootstrap trees were concatenated into a single file containing the 1000 bootstrap trees.

The ML phylogenetic trees were used for estimating the 'Temporal signal' by generating a linear regression of phylogenetic root-to-tip distances against the sampling dates using Tempest (v1.5 (*15*)). All subsets demonstrated a positive correlation between root-to-tip distances and sampling dates in all subsets (R^2=0.21-0.27).

Model selection

A single subset (subset 2) was arbitrarily selected for the model selection process (including the discrete trait model selection) and the final model combination was applied to all ten subsets.

A general time reversible (GTR) substitution model was selected using Jmodeltest (v2.1.10) (*16*,*17*). Different combinations of molecular clocks (i.e. strict or uncorrelated lognormal relaxed) and population growth models [i.e., constant, logistic, exponential, or Gaussian Markov random field (GMRF) Bayesian skyride] were evaluated. Each model combination was tested in two independent Markov chain-Monte Carlo (MCMC) runs of at least 200 million iterations, with sampling every 20,000 iterations. Convergence and proper mixing of all MCMC runs (ensuring an effective sample size > 200) and agreement between the independent MCMC runs for each model were verified manually using Tracer (v1.7.1 (*18*)) after excluding 10% of the MCMC run as burn-in. The log marginal likelihoods for each model were obtained using path sampling (PS)/stepping-stone sampling (SS) (see below) and the models were compared by calculating the Bayes factors (*19*,*20*). The combination of GTR substitution rates, uncorrelated lognormal relaxed molecular clock, and exponential growth demographic model had the highest log Bayes factor value and was chosen as the model specification for further analyses.

In addition to the ‘temporal signal’ analysis with Tempest (above), at this stage, a tip-date randomization test was conducted (with the selected model combination) using the package ‘TipDatingBeast’ (v1.0.6 (*21*)) in R (v3.4.3 (*22*)), and the evaluated time to most recent common ancestor (TMRCA) and mutation rate values were compared between the real data and the randomized trials (n=20). This further support the temporal structure of the data as no overlap was found between the highest posterior density $(HPD)_{95\%}$ credible intervals and mean values of the randomized trials (n=20) and real data, respectively, for time to the most recent common ancestor (TMRCA) and mutation rates (Appendix 2 Figure 13).

Discrete trait analysis

Bayesian phylogeographic reconstruction was conducted by incorporating the ‘collection location’ trait under a discrete trait geospatial model (*23*) using the model combination found in the previous step. Symmetric and asymmetric models for trait transition were considered. Each model combination was tested as described above. The combination of GTR substitution rates, uncorrelated lognormal relaxed molecular clock, and exponential growth with asymmetric trait transitions was selected in this step.

This model combination was then used with all remaining subsets (at least two independent runs of 200 million iterations or more were run for each). The directionality of the spatial diffusion process was examined by calculating the ‘collection location’ Markov jumps (estimates the expectations for state transitions from USA to Europe and from Europe to USA) and Markov rewards (waiting times; estimate the expectations for time that is spent in each location, Europe or USA) (*24*,*25*). We found that the transmission was predominantly unidirectional, as reflected by the high number of Markov jumps from Europe to USA and the long waiting periods (Markov rewards) in Europe (see text).

In all analyses, the independent MCMC run outputs (log and trees files) of each model were combined using LogCombiner (v1.8.4 (*3*)), after exclusion of a 10% burn-in period. TreeAnnotator (v1.8.4 (*3*)) was used for selection and annotation of a single maximum clade credibility tree (using common ancestor node heights).

In all models, a mutation rate of 3.4×10^{-7} substitutions per site per year, previously estimated for *S*. Typhimurium DT104 (*26*), was set as the mean value for a lognormal prior distribution of the clock rate (with standard deviation (stdev)=2). In addition, a lognormal

distribution (mean=10 and stdev=2.148) was set as prior population size. In addition to the priors described above, for logistic population growth models a Laplace distribution (mean=0, stdev=0.026568; initial value=0.26) was set as a prior. In order to avoid the potential ascertainment bias as only the variable site alignments were used in the analysis (ranged between 1195 and 1465 sites in the ten subsets), the total number of nucleotides in the reference genome (RefSeq NZ_LN999997; 1204735, 1315079, 1312718 and 1204706 for A, C, G & T, respectively) were incorporated in the xml file as an approximation of the non-variable sites in the analysis. The following text:

```
<patterns id="patterns" from="1" strip="false">
        <alignment idref="alignment"/>
    </patterns>
```

Was replaced with:

```
<mergePatterns id="patterns">
 <patterns from="1" every="1">
 <alignment idref="alignment"/>
 </patterns>

 <constantPatterns>
 <alignment idref="alignment"/>
 <counts>
 <parameter value="1204735 1315079 1312718 1204706"/>
 </counts>
 </constantPatterns>
 </mergePatterns>
```

In addition, for estimating the time of the main introduction into USA, the clade’s ancestral node was visually identified in each of the ten subsets maximum clade credibility trees and the node height and high posterior density 95% ($HPD_{95\%}$) were recorded and the average value for all subsets were calculated (Appendix 2 Table 1 and Figures 1-10) .

Moreover, the analysis of subset 2 (without the discrete trait analysis) was repeated (with two independent seeds) while sampling only from the prior, to verify that prior parameters were not over-constraining the calculations.

In all models, The log marginal likelihoods for the model were obtained using path sampling (PS) / stepping-stone sampling (SS) (for both PS and SS - 100 paths were used, and the number of PS/SS iterations was equal to number of MCMC iterations in the analysis divided by the number of PS/SS paths) and the models were compared by calculating the Bayes factors (*19*,*20*).

Association-index (AI)

Evaluation and quantification of the correlation between the location trait and shared ancestry was done using the Bayesian Tip-association Significance testing (BaTS v0.9.0) (*27*) software. Briefly, the software calculates the AI for each tree in the posterior distribution of the real data and for permutations in which the traits are randomly assigned to the tree tips. The latter will serve as the null hypothesis, in which there is no association between the trait and ancestry. A posterior distribution of AI statistic is formed for the real data and for the permutations. The test significance is obtained by calculating the proportion of the mean posterior estimate for the permutations that are extreme than the mean posterior for the real data. The trait-ancestry correlation was estimated (using 1,000 permutations) for each of the ten subsets in this analysis and found statistically significant ($p<0.05$; Appendix 2 Table 2) with an average ratio of 0.12 ($HPD_{95\%}$: 0.11-0.14), suggesting an association between the sampling locations and the tree phylogeny.

Potential additional introductions

In addition to the main introduction from Europe to USA, the maximum clade credibility trees of all subsets were visually scanned to detect a few sequences (up to five in a monophyletic group) intermingled with sequences from a different location (i.e. European sequences located within USA main clade and/or USA sequences that were not part of USA main clade; Appendix 2 Figures 1-10), which can potentially indicate additional introductions. Sequence details were summarized and further investigation of potential travel history records was conducted. Overall, 48 sequences (6 from Europe and 42 from USA) that represent potential additional introductions (between 4 and 14 sequences were included in each subset – Appendix 2, Table 4) were

identified. The six European sequences were recovered from human sources and 28, 5, 6 and 3 of USA sequences were recovered from humans, food products, livestock and other sources, respectively. History of recent international travel information was obtained for 22/28 of USA human isolates, of which information was not available for six (cases were not interviewed), 14 had not traveled recently and two had recently traveled: one became ill while traveling to the Philippines and the other was travelling to France for three days in the week before showing clinical signs (personal communication - Minnesota Department of Health).

Sequence typing and acquired antimicrobial resistance genes

De novo genome assemblies were constructed using SPAdes (v3.12.0 (*5*)). Acquired antimicrobial resistance genes (AARGs) and Multi Locus Sequence types (MLST) were identified using ResFinder (v2.1 (*28*)) and MLST; v1.6 (*29*)) tools from the 'bacterial analysis pipeline' in the Center for Genomic Epidemiology server (https://cge.cbs.dtu.dk/services/cge/). All tools and their databases were downloaded and installed on a local server (March 2018) and used with the 'bacterial analysis pipeline' default settings. Outcomes were combined in a tab-separated file using python (www.python.org) pandas package (v0.23.4 (*30*)).

The simultaneous presence of genotypic resistance to ampicillin, streptomycin, sulfonamides, and tetracycline (ASSuT) was defined according to Elnekave et al. (*1*) as simultaneous presence of bla_{TEM-1}, *strA*, *strB*, *sul2*, and *tet(B)* genes. However, the nomenclature of the genes in ResFinder was updated and therefore: *strB* gene was identified as *aph (6)-Id* gene; and *strA* gene was identified as *strA* or *aph(3'')-Ib* genes.

R (v3.4.3 (*22*)) was used to perform Pearson's chi-square or Fisher's exact tests [using package hypergea (v1.3.6 (*31*)) to incorporate the Haldane-Anscombe correction for zero values, when needed] for estimating associations between 'collection location', defined as USA or Europe, and: (i) the simultaneous presence of genotypic resistance to ASSuT; (ii) presence of predominant (found in at least 15 sequences) AARGs to quinolones; or iii) to extended spectrum cephalosporins (ESC; see text).

Data summarizing and visualization

The R packages 'dplyr' (v0.7.4 (*32*)), 'stringr' (v1.2.0 (*33*)) and 'tidyr' (v0.8.0 (*34*)) were used for data filtering and subset selection. Packages 'vioplot' (v0.3.0 (*35*)), 'HDInterval' (v0.2.0 (*36*)), 'ggplot2' (v2.2.1 (*37*)), 'gridExtra' (v2.3 (*38*)), 'ggpubr' (v0.2 (*39*)), 'lubridate' (v1.7.1 (*40*)) and 'ggtree' (v1.10.5 (*41*)) were used for output summary and visualization.

Phylogenetic ML tree reconstruction for the entire study population

In addition to the described analysis, to estimate whether the selected subsets are providing a good representation of the entire study population tree topology, a ML core genome phylogeny was reconstructed using all study isolates (n=1431). Core and pan genomes were obtained using the annotated [using Prokka (v1.13.3 (*42*))] assembled contigs in 'Roary' (v3.12.0 (*43*)) with an identity cut-off of 95%. Core genome ML tree reconstruction was conducted using RAxML (v8.2.10 (*14*)) (after removal of duplicates only 1185 sequences were included in the phylogeny tree; 25,687 variable sites were used for this analysis; Appendix 2, Figure 11) with a general time reversible substitution evolutionary model with gamma correction (GTR+Γ) and 1000 bootstrap replicates. Trees were rooted using *S*. Enteritidis isolates (SRR1965760 and SRR3242211). Packages 'ape' (v5.0 (*44*)) and 'ggtree' (v1.10.5 (*45*)) in R software (v3.4.3 (*22*)) were used for visualization.

Data availability

The raw XML and log files are available from the authors upon request.

References

1. Elnekave E, Hong S, Mather AE, Boxrud D, Taylor AJ, Lappi V, et al. Salmonella enterica Serotype 4,[5],12:i:- in Swine in the United States Midwest: An Emerging Multidrug-Resistant Clade. Clin Infect Dis. 2018;66:877–85. PubMed https://doi.org/10.1093/cid/cix909

2. Mather AE, Phuong TLT, Gao Y, Clare S, Mukhopadhyay S, Goulding DA, et al. New Variant of Multidrug-Resistant *Salmonella enterica* Serovar Typhimurium Associated with Invasive Disease in Immunocompromised Patients in Vietnam. MBio. 2018;9:e01056-18. PubMed https://doi.org/10.1128/mBio.01056-18

3. Drummond AJ, Rambaut A. BEAST: Bayesian evolutionary analysis by sampling trees. BMC Evol Biol. 2007;7:214. PubMed https://doi.org/10.1186/1471-2148-7-214

4. Andrews S. FastQC: a quality control tool for high throughput sequence data. 2010.

5. Bankevich A, Nurk S, Antipov D, Gurevich AA, Dvorkin M, Kulikov AS, et al. SPAdes: a new genome assembly algorithm and its applications to single-cell sequencing. J Comput Biol. 2012;19:455–77. PubMed https://doi.org/10.1089/cmb.2012.0021

6. Bushnell B. BBMap short-read aligner, and other bioinformatics tools.

7. Gurevich A, Saveliev V, Vyahhi N, Tesler G. QUAST: quality assessment tool for genome assemblies. Bioinformatics. 2013;29:1072–5. PubMed https://doi.org/10.1093/bioinformatics/btt086

8. Yoshida CE, Kruczkiewicz P, Laing CR, Lingohr EJ, Gannon VP, Nash JH, et al. The Salmonella In Silico Typing Resource (SISTR): An Open Web-Accessible Tool for Rapidly Typing and Subtyping Draft Salmonella Genome Assemblies. PLoS One. 2016;11:e0147101. PubMed https://doi.org/10.1371/journal.pone.0147101

9. Langmead B, Salzberg SL. Fast gapped-read alignment with Bowtie 2. Nat Methods. 2012;9:357–9. PubMed https://doi.org/10.1038/nmeth.1923

10. Li H, Handsaker B, Wysoker A, Fennell T, Ruan J, Homer N, et al.; 1000 Genome Project Data Processing Subgroup. The Sequence Alignment/Map format and SAMtools. Bioinformatics. 2009;25:2078–9. PubMed https://doi.org/10.1093/bioinformatics/btp352

11. Koboldt DC, Zhang Q, Larson DE, Shen D, McLellan MD, Lin L, et al. VarScan 2: somatic mutation and copy number alteration discovery in cancer by exome sequencing. Genome Res. 2012;22:568–76. PubMed https://doi.org/10.1101/gr.129684.111

12. Arndt D, Grant JR, Marcu A, Sajed T, Pon A, Liang Y, et al. PHASTER: a better, faster version of the PHAST phage search tool. Nucleic Acids Res. 2016;44(W1):W16-21. PubMed https://doi.org/10.1093/nar/gkw387

13. Croucher NJ, Page AJ, Connor TR, Delaney AJ, Keane JA, Bentley SD, et al. Rapid phylogenetic analysis of large samples of recombinant bacterial whole genome sequences using Gubbins. Nucleic Acids Res. 2015;43:e15. PubMed https://doi.org/10.1093/nar/gku1196

14. Stamatakis A. RAxML version 8: a tool for phylogenetic analysis and post-analysis of large phylogenies. Bioinformatics. 2014;30:1312–3. PubMed https://doi.org/10.1093/bioinformatics/btu033

15. Rambaut A, Lam TT, Max Carvalho L, Pybus OG. Exploring the temporal structure of heterochronous sequences using TempEst (formerly Path-O-Gen). Virus Evol. 2016;2:vew007. PubMed https://doi.org/10.1093/ve/vew007

16. Darriba D, Taboada GL, Doallo R, Posada D. jModelTest 2: more models, new heuristics and parallel computing. Nat Methods. 2012;9:772. PubMed https://doi.org/10.1038/nmeth.2109

17. Guindon S, Gascuel O. A simple, fast, and accurate algorithm to estimate large phylogenies by maximum likelihood. Syst Biol. 2003;52:696–704. PubMed https://doi.org/10.1080/10635150390235520

18. Rambaut A, Drummond AJ, Xie D, Baele G, Suchard MA. Posterior Summarization in Bayesian Phylogenetics Using Tracer 1.7. Syst Biol. 2018;67:901–4. PubMed https://doi.org/10.1093/sysbio/syy032

19. Baele G, Lemey P, Bedford T, Rambaut A, Suchard MA, Alekseyenko AV. Improving the accuracy of demographic and molecular clock model comparison while accommodating phylogenetic uncertainty. Mol Biol Evol. 2012;29:2157–67. PubMed https://doi.org/10.1093/molbev/mss084

20. Baele G, Li WL, Drummond AJ, Suchard MA, Lemey P. Accurate model selection of relaxed molecular clocks in bayesian phylogenetics. Mol Biol Evol. 2013;30:239–43. PubMed https://doi.org/10.1093/molbev/mss243

21. Rieux A, Khatchikian CE. tipdatingbeast: an r package to assist the implementation of phylogenetic tip-dating tests using beast. Mol Ecol Resour. 2017;17:608–13. PubMed https://doi.org/10.1111/1755-0998.12603

22. R Core Team. R: A language and environment for statistical computing. R Foundation for Statistical Computing. Vienna, Austria; 2016.

23. Lemey P, Rambaut A, Drummond AJ, Suchard MA. Bayesian phylogeography finds its roots. PLOS Comput Biol. 2009;5:e1000520. PubMed https://doi.org/10.1371/journal.pcbi.1000520

24. Minin VN, Suchard MA. Counting labeled transitions in continuous-time Markov models of evolution. J Math Biol. 2008;56:391–412. PubMed https://doi.org/10.1007/s00285-007-0120-8

25. Minin VN, Suchard MA. Fast, accurate and simulation-free stochastic mapping. Philos Trans R Soc Lond B Biol Sci. 2008;363:3985–95. PubMed https://doi.org/10.1098/rstb.2008.0176

26. Mather AE, Reid SW, Maskell DJ, Parkhill J, Fookes MC, Harris SR, et al. Distinguishable epidemics of multidrug-resistant Salmonella Typhimurium DT104 in different hosts. Science. 2013;341:1514–7. PubMed https://doi.org/10.1126/science.1240578

27. Parker J, Rambaut A, Pybus OG. Correlating viral phenotypes with phylogeny: accounting for phylogenetic uncertainty. Infect Genet Evol. 2008;8:239–46. PubMed https://doi.org/10.1016/j.meegid.2007.08.001

28. Zankari E, Hasman H, Cosentino S, Vestergaard M, Rasmussen S, Lund O, et al. Identification of acquired antimicrobial resistance genes. J Antimicrob Chemother. 2012;67:2640–4. PubMed https://doi.org/10.1093/jac/dks261

29. Larsen MV, Cosentino S, Rasmussen S, Friis C, Hasman H, Marvig RL, et al. Multilocus sequence typing of total-genome-sequenced bacteria. J Clin Microbiol. 2012;50:1355–61. PubMed https://doi.org/10.1128/JCM.06094-11

30. McKinney W. Data structures for statistical computing in python. Proceedings of the 9th Python in Science Conference; 2010: van der Voort S, Millman J; 2010. p. 51-6.

31. Boenn M. hypergea: Hypergeometric Tests. R package version 1.3.6. https://CRAN.R-project.org/package=hypergea; 2018.

32. Wickham H, Francois, R., Henry, L., Müller, K. dplyr: A Grammar of Data Manipulation. R package version 0.7.4. 2017.

33. Wickham H. stringr: Simple, Consistent Wrappers for Common String Operations. 2015 [cited; Available from: https://CRAN.R-project.org/package=stringr

34. Wickham H, Henry L. tidyr: Easily Tidy Data with 'spread()' and 'gather()' Functions. R package. 0.8.0 ed; 2018.

35. Adler D, Kelly ST. vioplot: violin plot. R package. 0.3.0 ed; 2018.

36. Meredith M, Kruschke J. HDInterval: Highest (Posterior) Density Intervals. R package 0.2.0 ed; 2018.

37. Wickham H. Ggplot2 : elegant graphics for data analysis. New York: Springer; 2009.

38. Auguie B. gridExtra: Miscellaneous Functions for "Grid" Graphics. R package version 2.3. 2017.

39. Kassambara A. ggpubr: 'ggplot2' Based Publication Ready Plots. R package version 0.2. 2018.

40. Grolemund G, Wickham H. Dates and times made easy with lubridate. J Stat Softw. 2011;40:1–25. https://doi.org/10.18637/jss.v040.i03

41. Yu G, Smith DK, Zhu H, Guan Y, Lam TTY. ggtree: an R package for visualization and annotation of phylogenetic trees with their covariates and other associated data. Methods Ecol Evol. 2017;8:28–36. https://doi.org/10.1111/2041-210X.12628

42. Seemann T. Prokka: rapid prokaryotic genome annotation. Bioinformatics. 2014;30:2068–9. PubMed https://doi.org/10.1093/bioinformatics/btu153

43. Page AJ, Cummins CA, Hunt M, Wong VK, Reuter S, Holden MT, et al. Roary: rapid large-scale prokaryote pan genome analysis. Bioinformatics. 2015;31:3691–3. PubMed https://doi.org/10.1093/bioinformatics/btv421

44. Paradis E, Claude J, Strimmer K. APE: Analyses of Phylogenetics and Evolution in R language. Bioinformatics. 2004;20:289–90. PubMed https://doi.org/10.1093/bioinformatics/btg412

45. Yu G, Smith DK, Zhu H, Guan Y, Lam TT-Y. ggtree: an r package for visualization and annotation of phylogenetic trees with their covariates and other associated data. Methods Ecol Evol. 2017;8:28–36. https://doi.org/10.1111/2041-210X.12628

Script 1 – Masking phage and recombinant regions

```
# filter_snps -r [regions file] -f [VCF file] -o [output folder]
# masks bases in a VCF file with locations specified in the regions file
import argparse
import pandas as pd
import os
def filter_phage(regions,pos,alt):
found = False
for i in range(len(regions)):
j=regions.ix[i]
if pos >= j.start and pos <= j.stop:
return 'N'
return alt
def main():
#argparse
parser = argparse.ArgumentParser()
```

```
parser.add_argument('-r','--regions',help='Input tab separated regions file (no header)',required=True)
parser.add_argument('-f','--file',help='Input vcf formatted SNPS file',required=True)
parser.add_argument('-o','--output',help='Output folder',required=True)
args=parser.parse_args()
outputfolder = args.output
vcfFile = args.file
regions = args.regions
name = vcfFile.split('/')[-1].split('.')[0]
#create output folder if it doesnt exist
if not os.path.exists(outputfolder):
os.mkdir(outputfolder)
regions=pd.read_csv(regions,sep='\t',names=['id','start','stop']).sort_values('start')
vcf = pd.read_csv(vcfFile,sep='\t',skiprows=23)
vcf['ALT']=vcf.apply(lambda row: filter_phage(regions,row['POS'], row['ALT']), axis=1)
with open(vcfFile) as myfile:
head = [next(myfile) for x in range (23)]
test=[]
test.append(vcfFile)
counts = vcf['ALT'].value_counts().to_dict()
#test.append(counts.get('A')+counts.get('T')+counts.get('C')+counts.get('G'))
print(counts)
with open(outputfolder+'/'+name+'_filtered.vcf','w') as newfile:
newfile.writelines(head)
```

```
vcf.to_csv(newfile,mode='a',sep='\t',index=False)

main()
```

Script 2 – Duplicates removal

```
import sys

from Bio import SeqIO

#clean.py [fasta file]

def sequence_cleaner(fasta_file, min_length=0, por_n=100):

# Create our hash table to add the sequences

sequences={}

# Using the Biopython fasta parse we can read our fasta input

for seq_record in SeqIO.parse(fasta_file, "fasta"):

# Take the current sequence

sequence = str(seq_record.seq).upper()

# Check if the current sequence is according to the user parameters

if (len(sequence) >= min_length and

(float(sequence.count("N"))/float(len(sequence)))*100 <= por_n):

# If the sequence passed in the test "is it clean?" and it isn't in the

# hash table, the sequence and its id are going to be in the hash

if sequence not in sequences:

sequences[sequence] = seq_record.id

# If it is already in the hash table, we're just gonna concatenate the ID

# of the current sequence to another one that is already in the hash table

else:

sequences[sequence] += "_" + seq_record.id
```

```
# Write the clean sequences
# Create a file in the same directory where you ran this script
output_file = open("clear_" + fasta_file, "w+")
# Just read the hash table and write on the file as a fasta format
for sequence in sequences:
output_file.write(">" + sequences[sequence] + "\n" + sequence + "\n")
output_file.close()
print("CLEAN!!!\nPlease check clear_" + fasta_file)
userParameters = sys.argv[1:]
try:
if len(userParameters) == 1:
sequence_cleaner(userParameters[0])
elif len(userParameters) == 2:
sequence_cleaner(userParameters[0], float(userParameters[1]))
elif len(userParameters) == 3:
sequence_cleaner(userParameters[0], float(userParameters[1]),
float(userParameters[2]))
else:
print("There is a problem!")
except:
print("There is a problem!")
```

Appendix 2 Table 1. The estimated time to the most recent common ancestor (TMRCA) of the main USA *S*. 4,[5],12:i:- ST34 clade in each subset. The ancestor node was visually identified* in each subset and an average value was calculated for all*

Subset	TMRCA - USA clade ($HPD_{95\%}$)
1	2005 (2002-2007)
2	2002 (1996-2007)
3	2006 (2004-2007)
4	2003 (2000-2006)
5	2003 (2001-2006)
6	2004 (2000-2007)
7	2006 (2004-2007)
8	2002 (1999-2005)
9	2001 (1995-2005)
10	2003 (2000-2006)
All (average)	2004 (2000-2006)

*The ancestor nodes are indicated in Appendix 2, Figures 1–10. $HPD_{95\%}$, highest posterior density 95% credible interval

Appendix 2 Table 2. The association index (AI) statistic mean of the real data and the randomized permutations* (the null hypothesis) for each of the ten subsets; and the AI ratio† between the real data and randomized permutations.

	Mean ($HPD_{95\%}$‡)			
Subset	Observed data	Null hypothesis (randomized permutations)	AI ratio	p value
1	0.49 (0.41-0.59)	5.95 (4.67-7.25)	0.08 (0.08-0.09)	0.0
2	0.37 (0.31-0.48)	5.91 (4.59-7.26)	0.06 (0.06-0.07)	0.0
3	0.48 (0.32-0.75)	5.36 (4.2-6.54)	0.09 (0.08-0.11)	0.0
4	0.68 (0.4-0.9)	5.75 (4.55-6.99)	0.12 (0.09-0.13)	0.0
5	0.76 (0.66-0.91)	6.06 (4.67-7.43)	0.13 (0.12-0.14)	0.0
6	0.62 (0.35-0.95)	6.19 (4.89-7.49)	0.1 (0.07-0.13)	0.0
7	0.99 (0.57-1.42)	5.69 (4.42-6.95)	0.17 (0.13-0.2)	0.0
8	0.58 (0.38-0.92)	6.03 (4.7-7.34)	0.1 (0.08-0.13)	0.0
9	0.96 (0.65-1.21)	6.07 (4.72-7.36)	0.16 (0.14-0.16)	0.0
10	1.28 (1.02-1.66)	6.19 (4.91-7.45)	0.21 (0.21-0.22)	0.0

*BaTS (v0. 9.0; (Appendix 2, ref. 26)) was used for calculating the AI and 1000 permutations were set in the analysis of each subset.
† AI mean of Real (observed) data / randomized permutations. Values closer to zero, rejects the null hypothesis (AI ratio = 1) and suggest association between location and ancestry (as was described by Mather et al. (Appendix 2, ref. 25))
‡ HPD95%= Highest posterior density 95% credible interval

Appendix 2 Table 3. The number sequences from potential additional introductions through travel/import identified in each subset*

	Collection location		
Subset	Europe	USA	Total
1	1	10	11
2		5	5
3		8	8
4	2	4	6
5		10	10
6	1	7	8
7	2	10	12
8		7	7
9		14	14
10		13	13
All	6	88	94

*Introductions in addition to the one leading to the main USA *S*. 4,[5],12:i:- ST34 clade (as described in the text); Some of the sequences are included in more than one subset. Overall, 48 unique sequences were identified as possible travel/import source for the additional introductions.

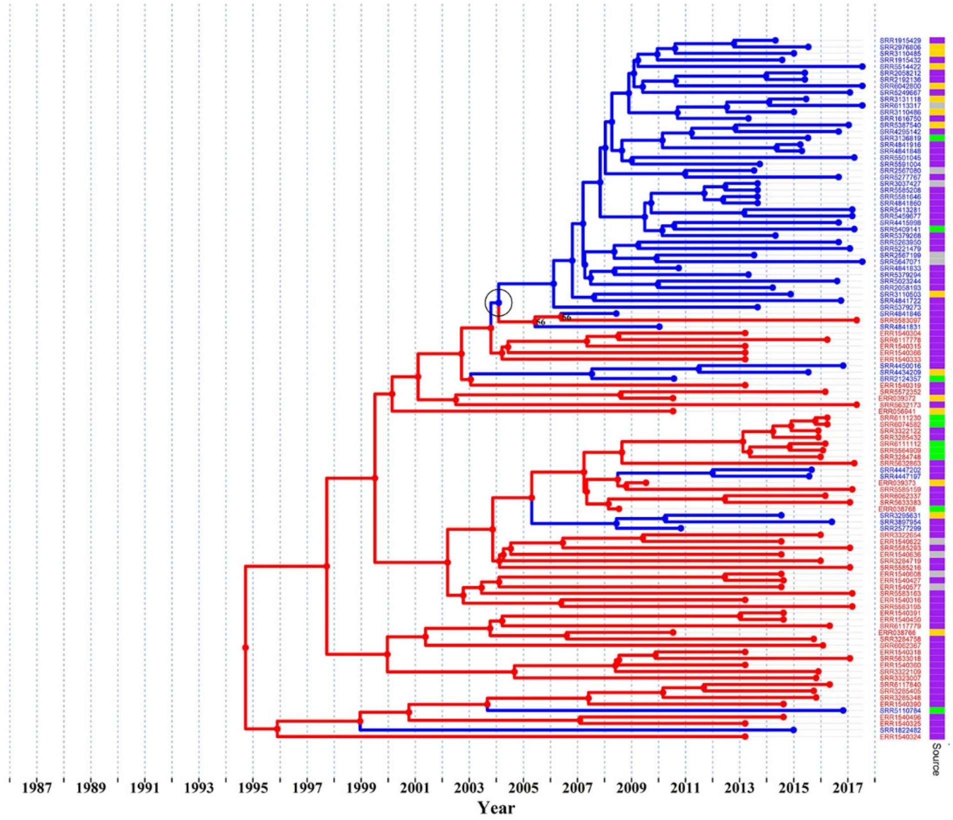
Source
1987 1989 1991 1993 1995 1997 1999 2001 2003 2005 2007 2009 2011 2013 2015 2017
Year

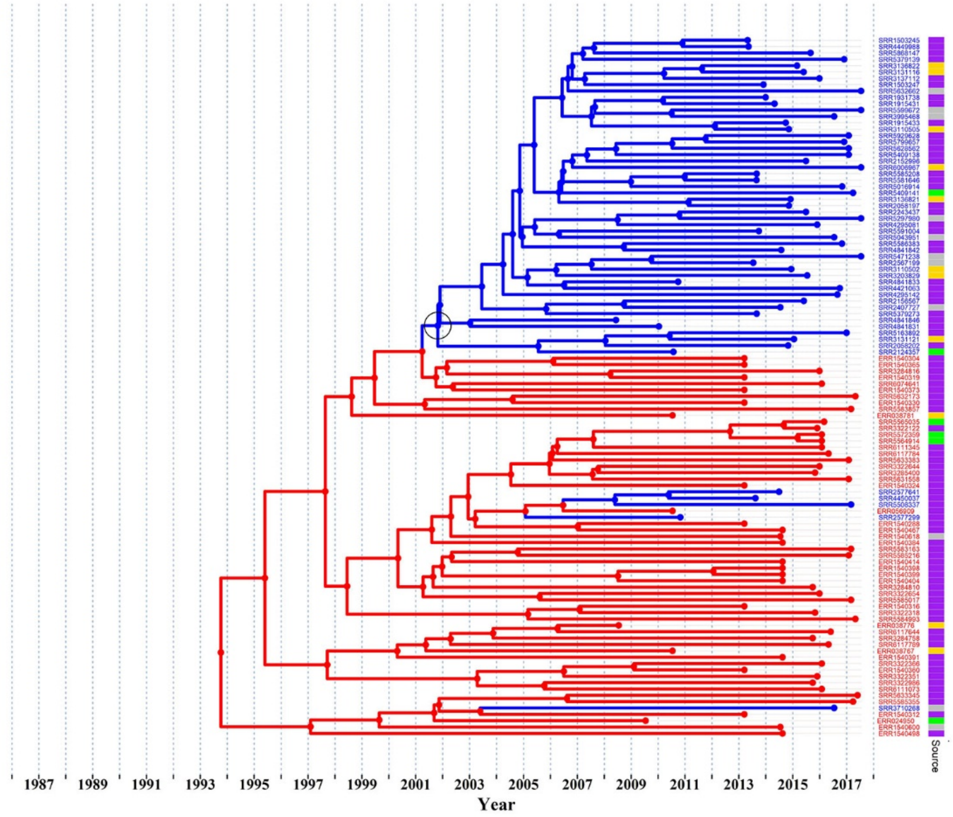
Source
1987 1989 1991 1993 1995 1997 1999 2001 2003 2005 2007 2009 2011 2013 2015 2017
Year

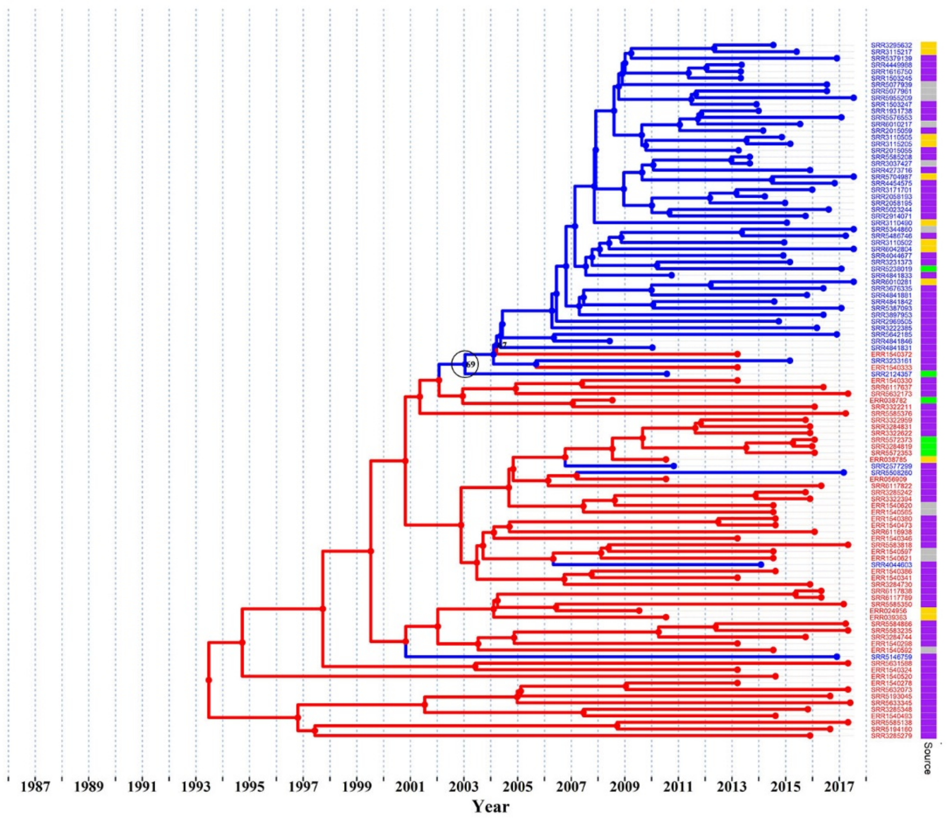
1987
1989
1991
1993
1995
1997
1999
2001
2003
2005
2007
2009
2011
2013
2015
2017
Year
Source

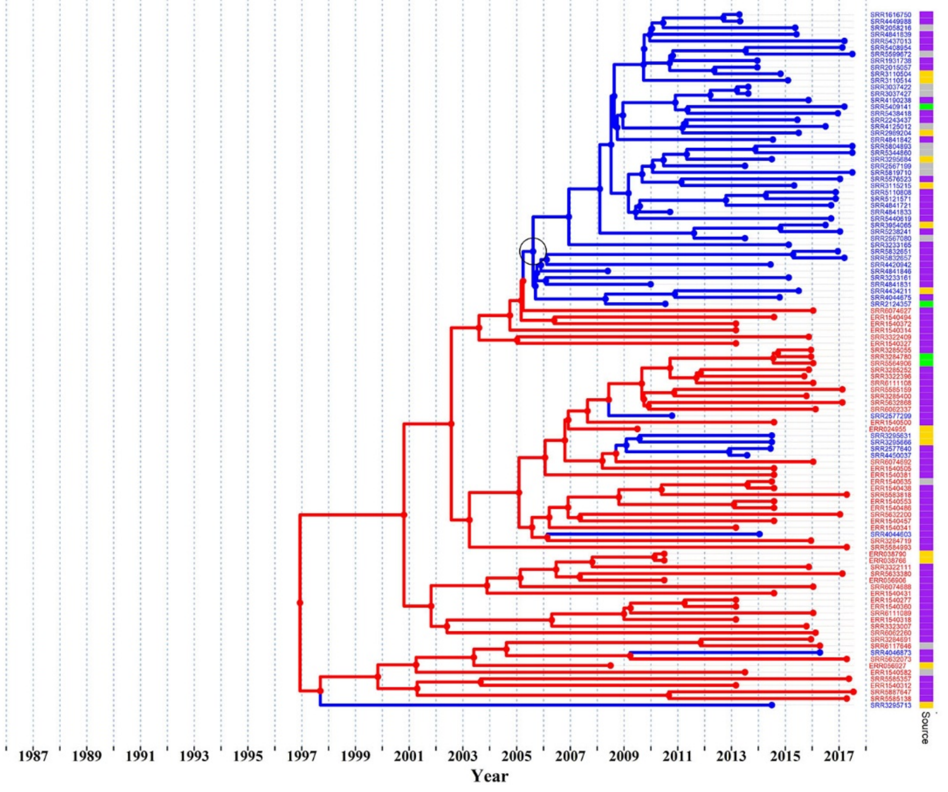
1987
1989
1991
1993
1995
1997
1999
2001
2003
2005
2007
2009
2011
2013
2015
2017
Year
Source

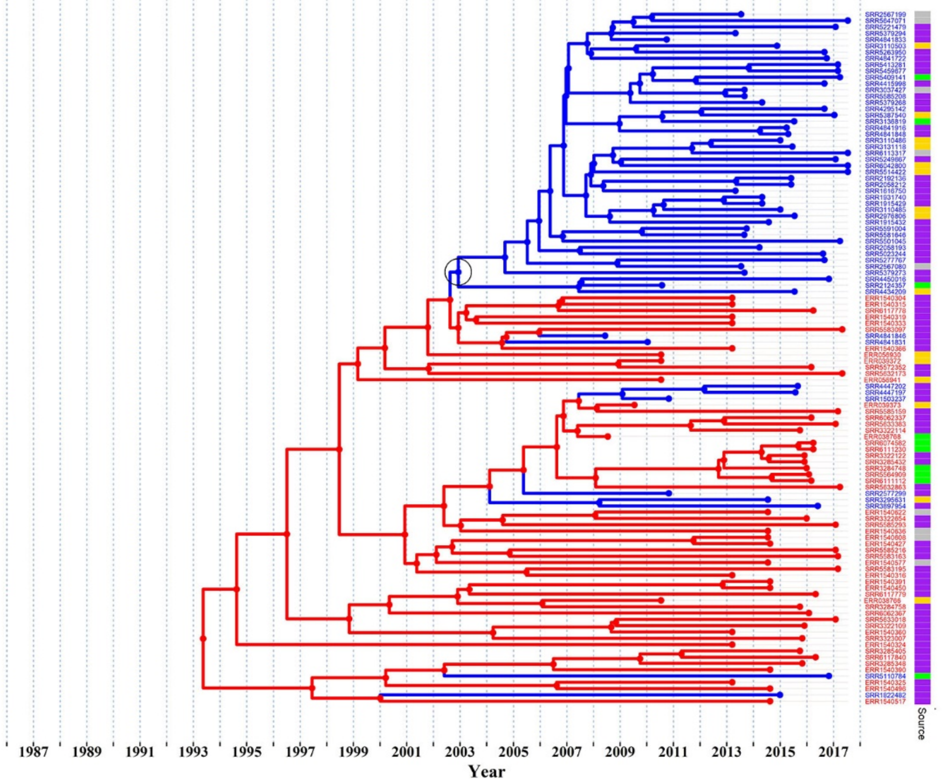
1987
1989
1991
1993
1995
1997
1999
2001
2003
2005
2007
2009
2011
2013
2015
2017
Year
Source

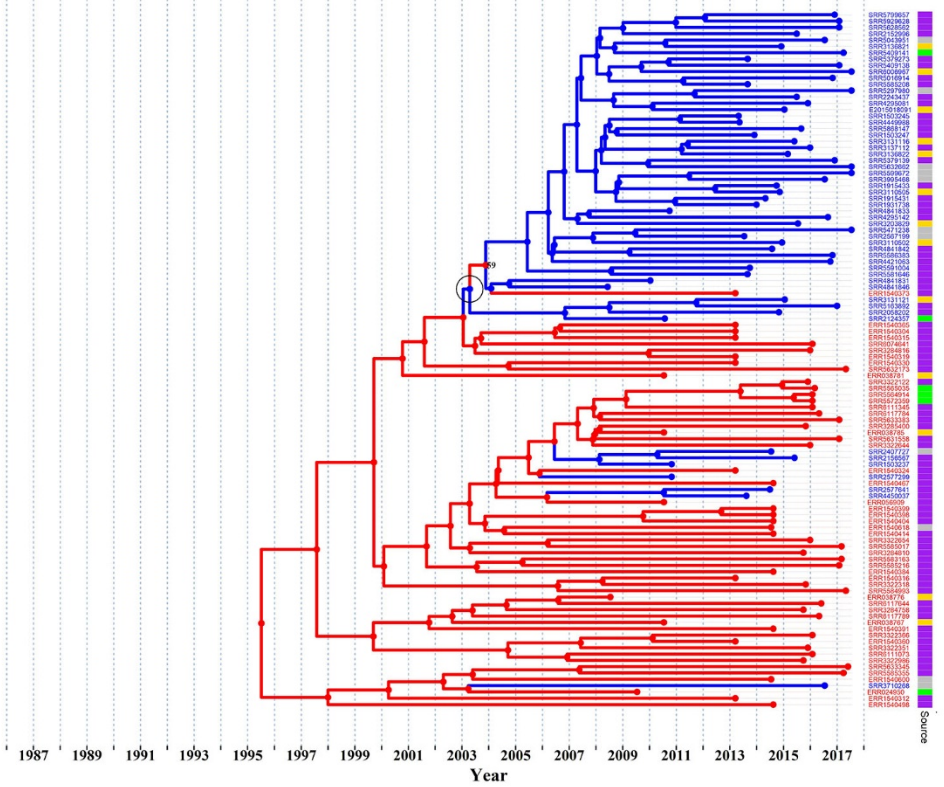
59
1987
1989
1991
1993
1995
1997
1999
2001
2003
2005
2007
2009
2011
2013
2015
2017
Year
Source

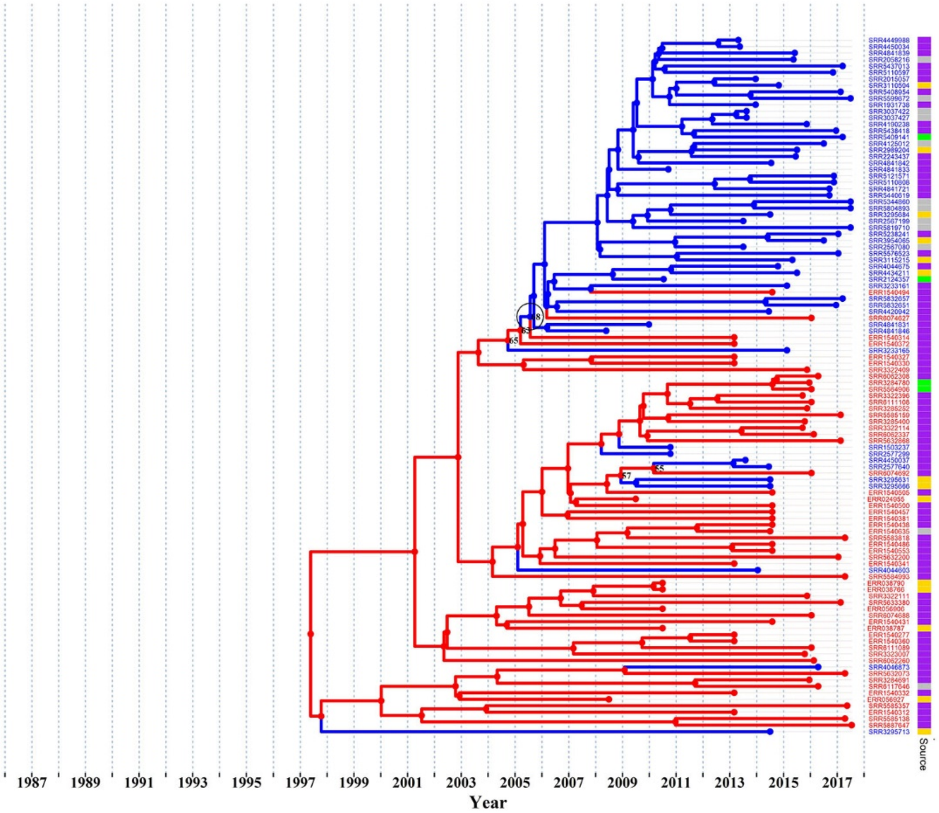
1987
1989
1991
1993
1995
1997
1999
2001
2003
2005
2007
2009
2011
2013
2015
2017
Year
Source

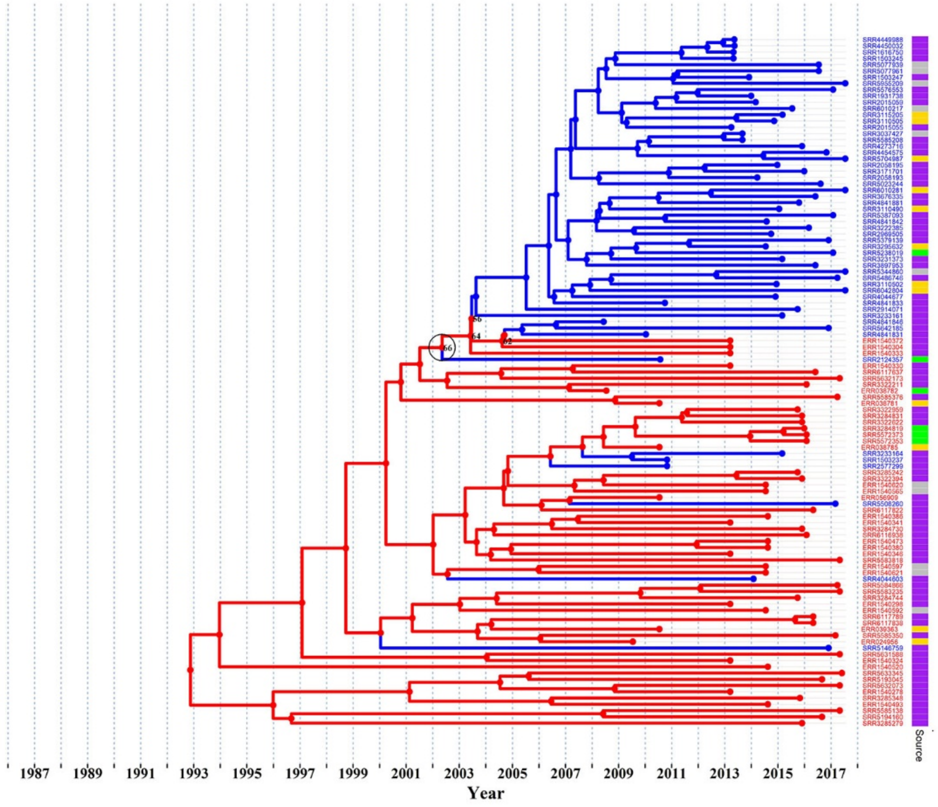
1987
1989
1991
1993
1995
1997
1999
2001
2003
2005
2007
2009
2011
2013
2015
2017
Year
Source

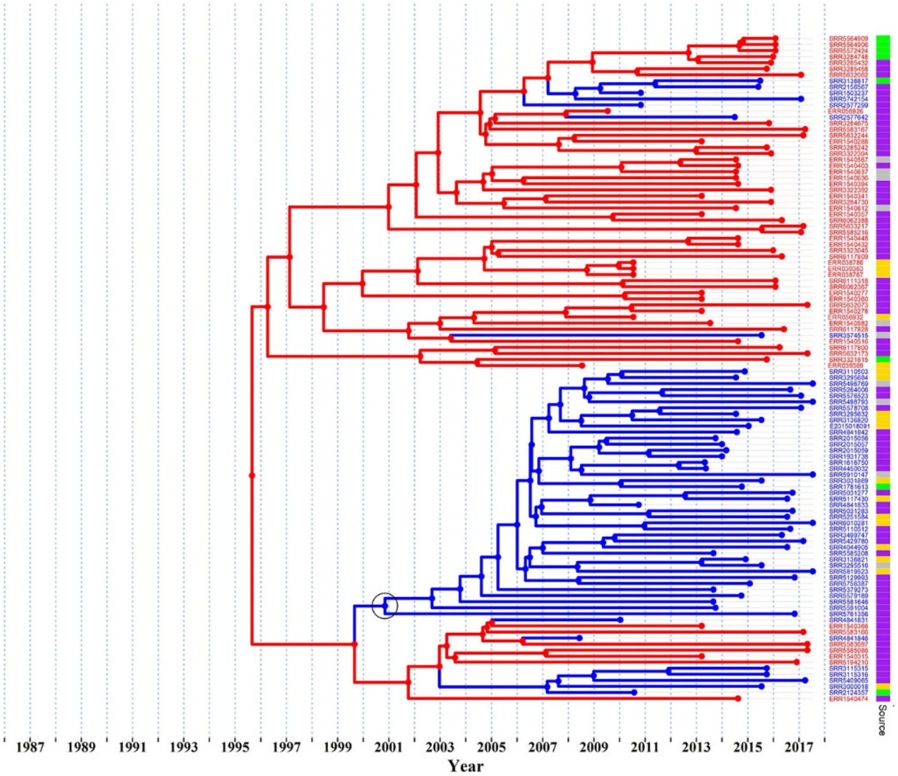
1987
1989
1991
1993
1995
1997
1999
2001
2003
2005
2007
2009
2011
2013
2015
2017
Year
Source

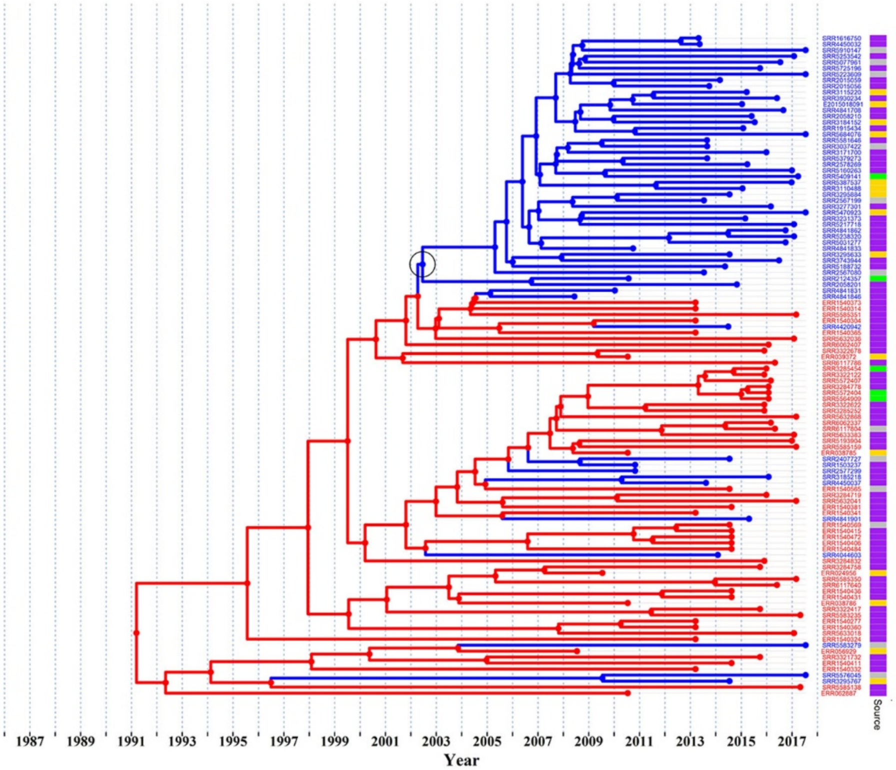
1987
1989
1991
1993
1995
1997
1999
2001
2003
2005
2007
2009
2011
2013
2015
2017
Year
Source

Appendix 2 Figures 1–10. Time scaled phylogenetic analysis of *S*. 4,[5],12:i:- ST34 isolates in subsets 1 to 10 (n ranged between 106 and 110 sequences following duplicates removal). Isolates were collected in USA (blue) and Europe (red) between 2008 and 2017 from multiple sources. Asymmetrical discrete trait analysis model was used to predict the locations on the nodes. The nodes, branches and tree tips were annotated according to the collection location. Nodes with location probabilities below 70% are indicated (with the location probability value). The ancestor node of the main USA *S*. 4,[5],12:i:- ST34 clade is circled (black). In addition, the bacterial isolate source [‘Food product’ (grey), ‘Human’ (purple), ‘Livestock’ (yellow) and ‘Other’ (green)] is depicted in the heatmap appended to the tree tips.

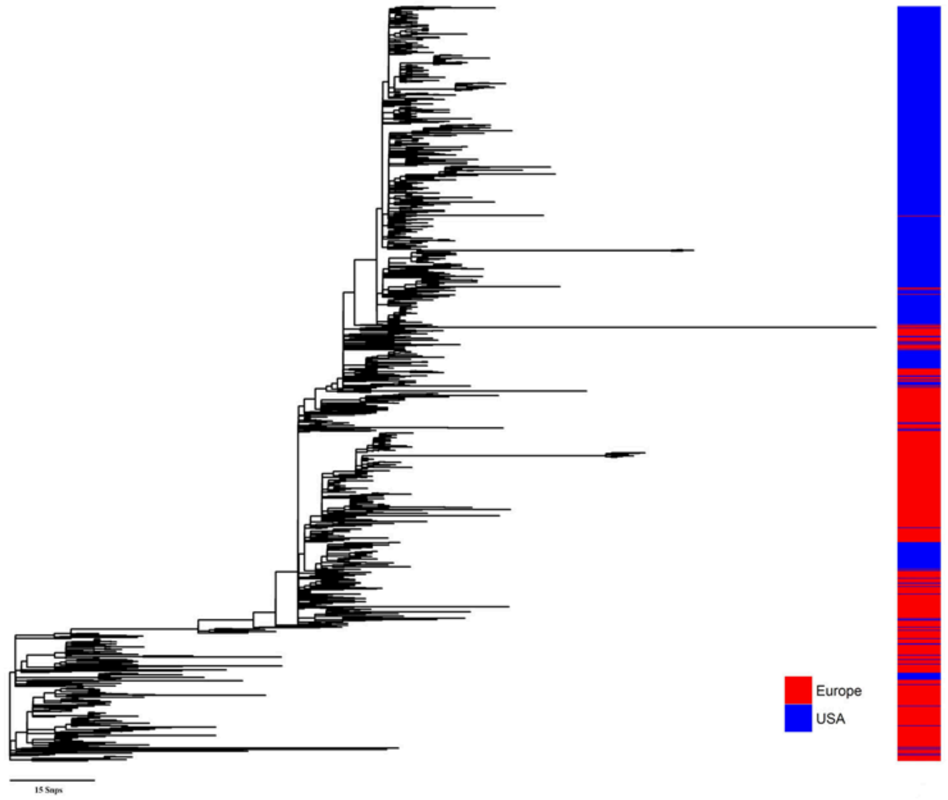

Appendix 2 Figure 11. A maximum likelihood phylogeny tree of the core genome of all *S*. 4,[5],12:i:- ST34 isolates (1431 sequences were included in the analysis, yet after removal of duplicates, 1185 sequences were included in the phylogeny tree; 25,687 variable sites were used for this analysis). Isolates were collected in USA (blue) and Europe (red) between 2008 and 2017 from multiple sources. *Salmonella* Enteritidis isolates (SRR1965760 and SRR3242211) were used as outgroup (not included in the figure).

A

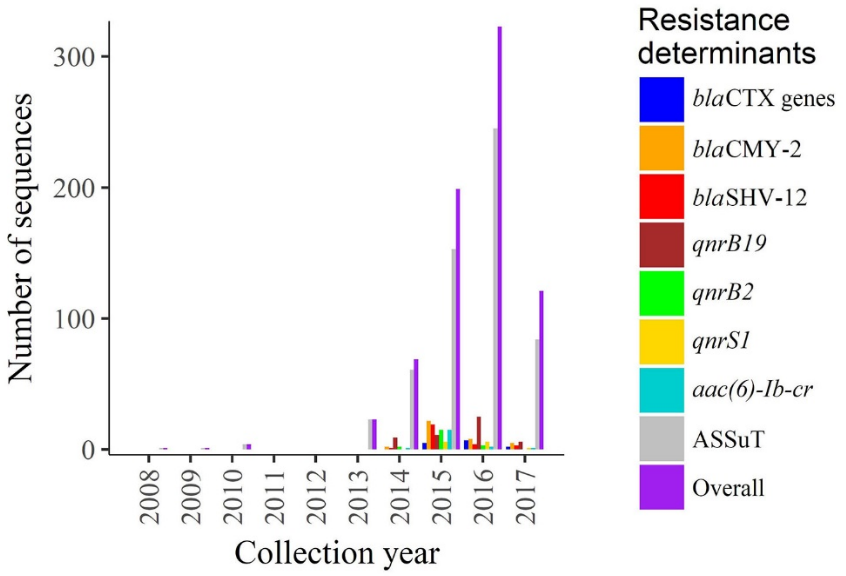

B

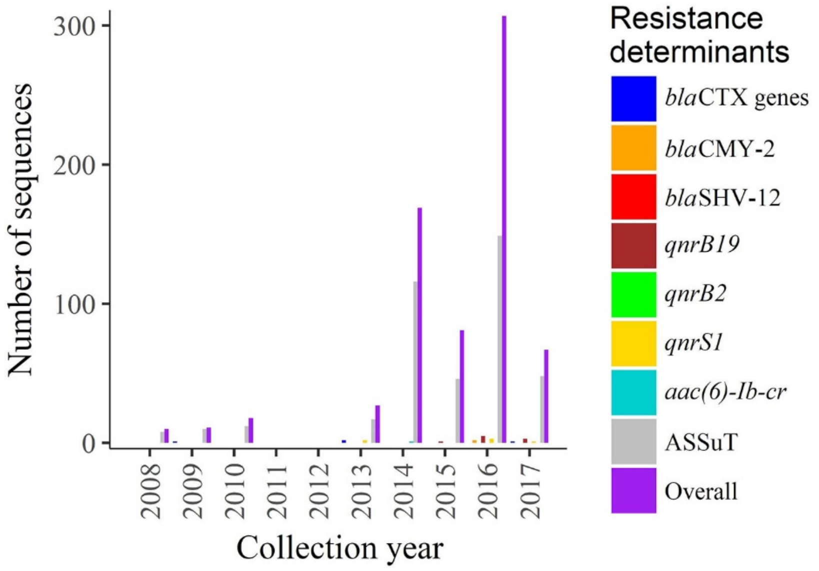

Appendix 2 Figure 12. The number of *S*. 4,[5],12:i:- ST34 sequences at the NCBI repository included in the study population and were collected in the USA (A) and Europe (B) in different years. The graph bars indicate of the overall number (purple) of available sequences and the number of sequences harboring genetic resistance determinants: *bla*$_{CTX-M}$ genes (blue), *bla*$_{CMY-2}$ (orange), *bla*$_{SHV-12}$ (red), *qnrB19* (brown), *qnrB2* (green), *qnrS1* (yellow), *aac(6')-Ib-cr* (turquoise) and ASSuT (grey).

A

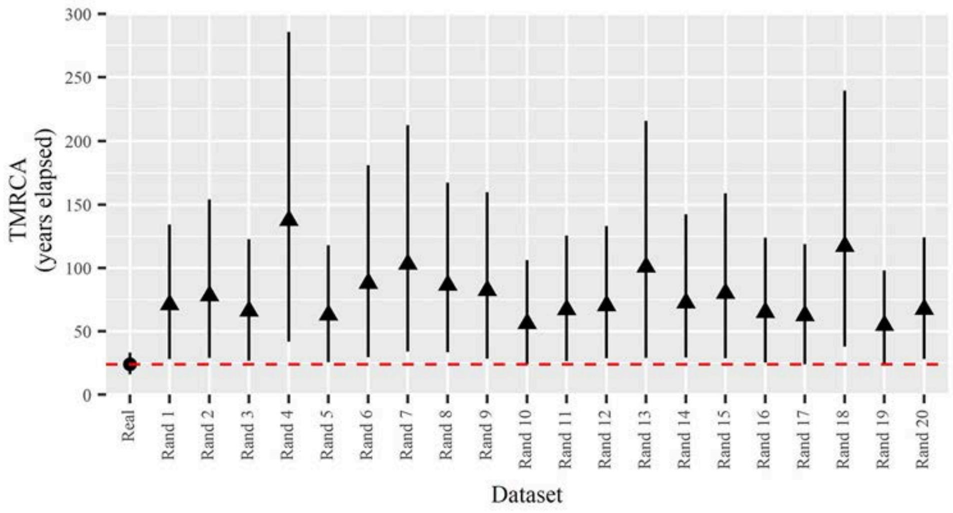

B

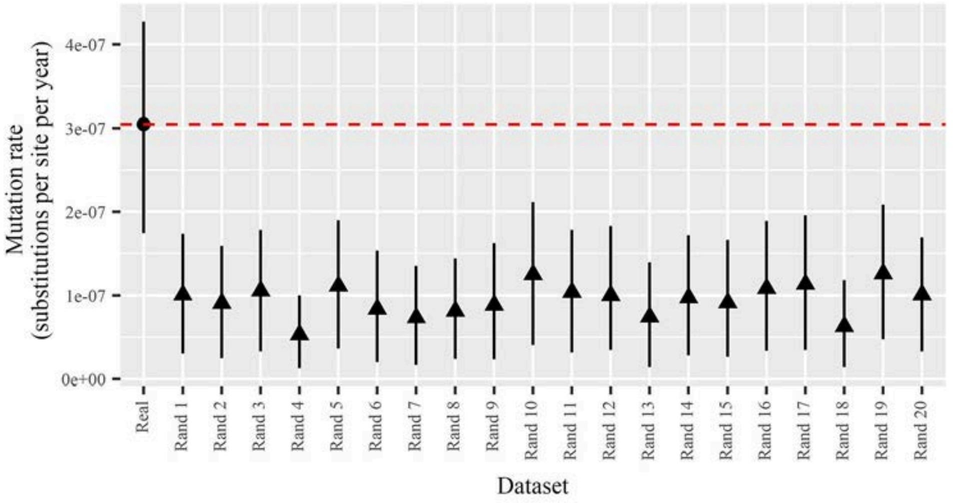

Appendix 2 Figure 13. The mean posterior values [with 95% highest posterior density (HPD) credible interval] of the mutation rate (upper inset) and the time to the most recent common ancestor (TMRCA; lower inset) parameters are indicated for the real data (circle) and the randomized date repeats (triangles). The mean values of the real data are further emphasized with dashed red lines.
